# Spinal Metastasis of Well-Differentiated Liposarcoma Component in Retroperitoneal Dedifferentiated Liposarcoma Treated by Minimally Invasive Surgery

**DOI:** 10.1155/2018/1708572

**Published:** 2018-07-16

**Authors:** Jiro Ichikawa, Tetsuro Ohba, Hiroaki Kanda, Koji Fujita, Shigeto Ebata, Hirotaka Haro

**Affiliations:** ^1^Department of Orthopaedic Surgery, Graduate School of Medicine, University of Yamanashi, 1110 Shimokato, Chuo, Yamanashi 409-3898, Japan; ^2^Department of Pathology, The Cancer Institute of the Japanese Foundation for Cancer Research (JFCR), 3-8-31 Ariake, Koto-ku, Tokyo 135-8550, Japan

## Abstract

**Case:**

Generally, well-differentiated liposarcoma (WDL) has recurrence potential but lacks metastatic potential. We present a rare case of spinal metastasis of WDL component in retroperitoneal dedifferentiated liposarcoma (DDL) treated by tumor curettage and L1 laminectomy followed by percutaneous pedicle screw fixation. Histological examination showed metastasis of the WDL component of DDL. The patient was ambulatory until death.

**Conclusion:**

To our knowledge, no case of spinal metastasis of WDL component in retroperitoneal DDL has been reported. We should carefully consider characteristics of DDLs during treatment. Minimally invasive surgery may be a powerful tool in patients with spinal metastasis.

## 1. Introduction

Retroperitoneal sarcomas (RPS) are rare, accounting for approximately 12% of all soft tissue sarcomas [1]. Liposarcoma is the most frequent histological subtype, and well-differentiated liposarcoma (WDL) and dedifferentiated liposarcoma (DDL) account for 90% of retroperitoneal liposarcomas [2]. Although WDL lacks metastatic potential, WDL that has differentiated into DDL has metastatic capacity [3]. We report the first case of spinal metastasis of WDL component in retroperitoneal DDL and successful treatment by minimally invasive surgery (MIS).

## 2. Case Presentation

**Figure 1:**
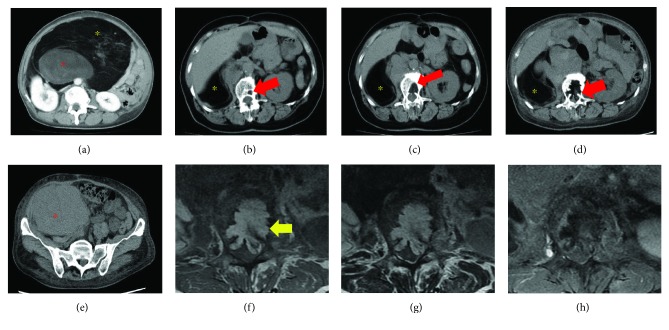
Abdominal computed tomography (CT). (a) Enhanced CT prior to the first surgery showed a large retroperitoneal mass in the second lumbar vertebra level, which consisted of both lipomatous (yellow asterisk) and nonlipomatous (red asterisk) components. Plain CT at 8 months (b) and 3 months (c) before our first visit showed metastatic lipomatous component (red arrow) involved in the vertebral body. (d, e) CT findings at our first visit showed both lipomatous (yellow asterisk) and nonlipomatous (red asterisk) components; in addition, the metastatic lipomatous component in the vertebral body had increased and destroyed the vertebral body. Magnetic resonance image of the lumbar spine. Axial T1-weighted (f), T2-weighted (g), and enhanced T1-weighted images (h) showed the mass with a similar intensity to fat and widespread from the vertebral body to the canal space (yellow arrow).

**Figure 2:**
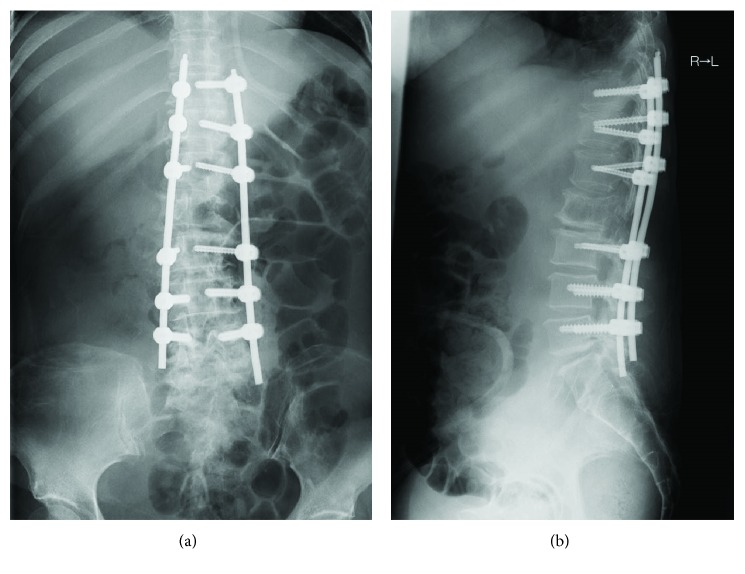
Postoperative radiograph of the anteroposterior view (a) and lateral view (b).

**Figure 3:**
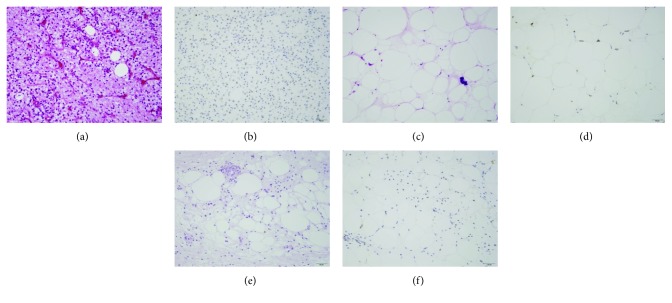
Histology of the primary site-dedifferentiated liposarcoma (a, b) and well-differentiated liposarcoma components (c, d) and the metastasis (e, f). (a, c, e) Hematoxylin-eosin stain. (b, d, f) Immunohistochemistry of MDM2. There was a mixed well-differentiated and dedifferentiated component in the primary lesion (a, c). Only the well-differentiated component was seen in the spine metastasis (e). Bar = 50 *μ*m.

A 65-year-old woman was admitted to our hospital because of low back pain and left posterior thigh and calf pain. When symptoms of sciatica began 2 months previously, she underwent radiography and magnetic resonance imaging (MRI) of the lumbar spine at another hospital. These showed a vertebral tumor in the lumbar spine. Both the patellar tendon and the Achilles tendon reflex were normal. The sensory exam was also normal. Although the left tibialis anterior (TA) muscle and extensor hallucis longus (EHL) muscle were manual muscle testing (MMT) grade 3, muscles other than the TA and EHL were MMT grade 5. Laboratory blood tests revealed hypoalbuminemia, anemia, and increased alkaline phosphatase and C-reactive protein. She had undergone resection of retroperitoneal DDL 5 years previously (Figure 1(a)) and repeated resection for recurrence 3 years previously. Recurrence occurred again 1 year previously, and spinal metastasis of WDL component occurred in the L2 vertebrae 8 months previously (Figure 1(b)) and gradually increased (Figure 1(c)) in computed tomography (CT), but she did not undergo additional treatment (Figures 1(d) and 1(e)). MRI showed a mass with high signal intensity on both T1-weighted images and T2-weighted images and no enhancement on gadolinium-enhanced T1-weighted images (Figures 1(f)–1(h)). The revised Tokuhashi score [4] was 11/15, and the Spinal Instability Neoplastic Score (SINP) was 10/18 [5]. Therefore, we diagnosed the vertebral tumor as the metastasis of WDL component in DDL and planned surgery for symptomatic improvement. Tumor curettage and L1 laminectomy followed by percutaneous pedicle screw fixation from the Th11 to L3 using intraoperative 3-D CT computer navigation were performed (Figures 2(a) and 2(b)). Histological examination showed mixed well-differentiated and well-dedifferentiated liposarcoma in the primary lesion (Figures 3(a), 3(c), and 3(e)). Lipoblasts containing hyperchromatic nuclei were apparent in the well-differentiated area. Myxoid liposarcoma was ruled out in the dedifferentiated area. Positive staining for MDM2 (Figures 3(b), 3(d), and 3(f)) and CDK4 (data not shown) by immunohistochemistry and negativity of *DDIT3* or *FUS* by FISH (data not shown) confirmed dedifferentiated liposarcoma. She could walk and had no pain in her back and no signs of palsy. However, the retroperitoneal mass subsequently increased, and she died 1.5 years after surgery.

## 3. Discussion

Histologically, liposarcoma is classified into four subtypes: well-differentiated, dedifferentiated, myxoid/round, and pleomorphic [6]. Evans et al. described DDL as high-grade and nonlipogenic sarcoma, juxtaposed with WDL [7]. Although the preferred site of myxoid, round, and pleomorphic liposarcoma is the extremities, WDL and DDL are common in the retroperitoneum, accounting for more than 90% of retroperitoneal liposarcomas [2, 8]. WDL and DDL show high-level amplification of CDK4 and MDM2, which are useful for differential diagnosis from other adipocytic tumors [9]. In our case, CDK4 and MDM2 expression in both the primary site and the metastatic site was confirmed by immunohistochemistry.

Histologically, WDL and DDL are quite different. WDL, which potentiates not metastasis but recurrence, is defined as an intermediate tumor, on the borderline of benign and malignant [6]. DDL generally shows high-grade sarcoma but can show low grade and a low ratio of dedifferentiation. Because the correlation of histological grade, ratio of dedifferentiation, and prognosis has been controversial, we should take care in making treatment decisions [10].

In the treatment of RPS, wide resection has been recommended and leads to better survival and local control [2, 11]. Wide resection is often impossible in RPS, because RPS have no characteristic symptoms; their size is much larger than the extremities, and they are usually found in important organs, including the kidney, colon, spleen, ureter, and common iliac artery and vein. However, wide resection often fails; the local 3- and 5-year recurrence rates are 31% and 47%, respectively. However, the 3- and 5-year survival rates of WDL are both 92%. In DDL, local recurrence rates at 3 and 5 years were reported as 43% and 60%, respectively [2, 8], and the survival rate was reported as 39% at 3 years and 61% at 5 years. Although the survival rate for DDL is much lower than that for WDL, it is higher than those for other sarcomas [12].

Adjuvant therapy, including chemotherapy and radiotherapy, should be considered because of the high incidence of recurrence and metastasis. Although the regimen of ifosfamide and doxorubicin has been reported in patients with DDL, the efficiency was low, and the development of new regimens and anticancer drugs is awaited [13]. Although radiotherapy and chemotherapy do not affect survival, both post- or preoperative radiotherapies can reduce recurrence, and preoperative radiotherapy is favored because of the low risk of radiation-induced toxicity [14]. Considering that our patient experienced recurrence twice, radiotherapy may have been advisable.

The number of patients with bone metastasis, including in the spine, has been increasing, because the number of patients with cancer has been increasing. Additionally, the prognosis of these patients has improved because of improvements in early diagnosis, surgery, chemotherapy, and radiotherapy. For surgical decisions about spinal metastasis, the revised Tokuhashi score has often been used, as has the recently developed SINP score. Although the revised Tokuhashi score is dependent on primary cancer type and patient condition, the SINP score is dependent on the stability of the spine, regardless of the primary cancer or patient condition. In our case, a revised Tokuhashi score of 11 indicated that treatment was dependent on the patient, and a SINP score of 10 indicated that the operation was favored because of spinal instability. Considering that our patient had only one metastasis and her muscle weakness and sciatica worsened, we decided to perform surgery. The option of MIS allowed us to opt for surgical treatment without the frequent accompanying adverse effects. We recently reported on the clinical efficacy and safety of minimally invasive percutaneous fixation surgery with intraoperative 3-D CT computer navigation [15]. Radiotherapy, including intensity-modulated radiation therapy and stereotactic radiosurgery, has been developed, such that the combination of MIS and radiotherapy has become standard [16]. In addition, the use of bone-modifying agents, including denosumab and zoledronate, may result in better local control.

While it is important to carefully evaluate the clinical behavior of the primary cancer as well as the patient's condition, the extent of surgical indication in spinal metastasis should be considered because of the development of multidisciplinary therapies and surgical techniques.
